# Femtosecond X-ray coherent diffraction of aligned amyloid fibrils on low background graphene

**DOI:** 10.1038/s41467-018-04116-9

**Published:** 2018-05-09

**Authors:** Carolin Seuring, Kartik Ayyer, Eleftheria Filippaki, Miriam Barthelmess, Jean-Nicolas Longchamp, Philippe Ringler, Tommaso Pardini, David H. Wojtas, Matthew A. Coleman, Katerina Dörner, Silje Fuglerud, Greger Hammarin, Birgit Habenstein, Annette E. Langkilde, Antoine Loquet, Alke Meents, Roland Riek, Henning Stahlberg, Sébastien Boutet, Mark S. Hunter, Jason Koglin, Mengning Liang, Helen M. Ginn, Rick P. Millane, Matthias Frank, Anton Barty, Henry N. Chapman

**Affiliations:** 1 Center for Free-Electron Laser Science, Deutsches Elektronen-Synchrotron, 22607 Hamburg, Germany; 2 The Hamburg Centre for Ultrafast Imaging, 22761 Hamburg, Germany; 3 Physics Department of the University of Zurich, 8057 Zurich, Switzerland; 4 Center for Cellular Imaging and NanoAnalytics (C-CINA), Biozentrum, University of Basel, 4058 Basel, Switzerland; 5 Lawrence Livermore National Laboratory, Livermore, CA 94550 USA; 6 Department of Electrical and Computer Engineering, University of Canterbury, Christchurch, 8140 New Zealand; 7 Department of Chemistry and Molecular Biology, 40530 Gothenburg, Sweden; 8 CNRS, CBMN UMR5248, IECB, Université de Bordeaux, 33600 Pessac, France; 9 Department of Drug Design and Pharmacology, University of Copenhagen, 2100 Copenhagen, Denmark; 10 Laboratory of Physical Chemistry, ETH Zürich, 8093 Zürich, Switzerland; 11 Linac Coherent Light Source, SLAC National Accelerator Laboratory, Menlo Park, CA 94025 USA; 12 Division of Structural Biology, The Wellcome Trust Centre for Human Genetics, University of Oxford, Roosevelt Drive Oxford, Oxfordshire, OX3 7BN UK; 13 Diamond House, Diamond Light Source, Harwell Science & Innovation Campus, Didcot, Oxfordshire OX11 0DE UK; 14 Department of Physics, University of Hamburg, 22761 Hamburg, Germany

## Abstract

Here we present a new approach to diffraction imaging of amyloid fibrils, combining a free-standing graphene support and single nanofocused X-ray pulses of femtosecond duration from an X-ray free-electron laser. Due to the very low background scattering from the graphene support and mutual alignment of filaments, diffraction from tobacco mosaic virus (TMV) filaments and amyloid protofibrils is obtained to 2.7 Å and 2.4 Å resolution in single diffraction patterns, respectively. Some TMV diffraction patterns exhibit asymmetry that indicates the presence of a limited number of axial rotations in the XFEL focus. Signal-to-noise levels from individual diffraction patterns are enhanced using computational alignment and merging, giving patterns that are superior to those obtainable from synchrotron radiation sources. We anticipate that our approach will be a starting point for further investigations into unsolved structures of filaments and other weakly scattering objects.

## Abstract (web summary)

The structures of amyloid fibres are currently primarily studied through solid state NMR and cryo-EM. Here the authors present a free-standing graphene support device that allows diffraction imaging of non-crystalline amyloid fibrils with single X-ray pulses from an X-ray free-electron laser.

## Introduction

High-resolution X-ray fiber diffraction is a key method for determining the structures of helical filaments that resist conventional crystallization^1, 2^. Helical structures consist of identical subunits, which repeat after a defined number of turns along the fiber axis. The diffraction pattern of such a helix, the Fourier transform of its electron density, is confined to layer lines^3^. The diffracted intensities on the layer lines can be used for structure determination as demonstrated for DNA, filamentous bacteriophages, and tobacco mosaic viruses^4–7^. However, not all filamentous systems with one-dimensional order yield diffraction patterns of a quality sufficient to infer a structure. Amyloid fibers consist of multiple protofibrils, are visibly polymorphic, and exhibit comparatively weak continuous diffraction in very few layer lines^8–10^. The sparse features in diffraction patterns from these fibers have so far provided, at best, constraints for low-resolution models or the validation of existing structural models^11–15^. Consequently, over the last few decades our knowledge about the structure of native amyloid fibrils has primarily been derived from other techniques including solid-state nuclear magnetic resonance (NMR)^16, 17^ and cryo-electron microscopy (cryo-EM)^18, 19^. However, these technologies have some limitations in dealing with these heterogeneous samples. High-resolution NMR structures depend on systems with very low polymorphism^20, 21^. NMR models give a local reconstruction of a small repeating unit of the fibril, and long-range packing or twists occurring in these fibrils can only be explored by cryo-EM. However, being able to image fibers, but not individual protofilaments, cryo-EM reconstructions represent averages of multiple fibril conformations. Protofibrils are the more relevant, disease-causing species found in equilibrium with mature fibers, and co-existing with different structured and unstructured assemblies^22^. X-ray free-electron laser (XFEL)-based experiments have the potential to record diffraction from individual protofibrils and build upon existing results from solid-state NMR and cryo-EM to improve our understanding of the structures of individual protofilaments.

Until now, the recording of high-resolution X-ray diffraction data from amyloid fibrils was limited by radiation damage, which destroys the specimen before meaningful diffraction can be recorded^23^. This loss of structure strongly depends on the total X-ray energy deposited in the sample per unit mass (the dose), which is itself proportional to the total X-ray fluence of the incident X-ray beam and thus the achievable diffraction signal. To mitigate this problem and obtain measurable diffraction patterns, the X-ray energy deposited per fiber is usually reduced by preparing a fiber specimen composed of millions of fibers mutually aligned along their fiber axes, which are simultaneously exposed to the X-ray beam with a lower flux^24–26^. For such a specimen, the scattering from the fiber is significantly amplified above the background levels from solvent and air. However, fibers mutually aligned in oriented bundles are usually randomly rotated about their fiber axes, giving a cylindrically averaged diffraction pattern of reduced information content. Furthermore, an average of polymorphic conformations are present in each diffraction pattern. This fact, together with deviations from perfect alignment, blurs details in the diffraction pattern.

XFELs extend the conventional dose limit by exposing the sample for only a few femtoseconds to intense X-ray pulses containing over 10^12^ quasi-monochromatic and spatially coherent photons that can be focused to a sub-micrometer spot. This allows a 'diffraction-before-destruction' approach, which enables the recovery of structural information before the photoelectron cascade destroys the molecules. At such high X-ray fluence, the conventional damage limit is increased, resulting in 10,000 times more scattered photons than is usually possible^27, 28^. Although this was originally proposed for single particle imaging^28^ and first implemented in the form of serial femtosecond crystallography (SFX) in 2009^29^, it has also recently been applied to imaging amyloid fibrils^30, 31^. Serial fiber diffraction at XFELs using a liquid jet delivery system has provided high-resolution data from a crystalline fiber system^31^. However, data quality for non-crystalline fibrils was poor^30^.

Non-crystalline amyloid protofibrils are often only a single-molecule thick and, therefore, about a thousand times smaller in width than the micrometer-thick water jet, the scattering of which, therefore, obscures their diffraction signal. To increase the achievable signal-to-noise ratio in fiber diffraction patterns, we have combined a new sample delivery medium based on free-standing graphene and the highly brilliant nanofocus XFEL beam of the Coherent X-ray Imaging (CXI) instrument^32^ at the Linac Coherent Light Source (LCLS). Ultraclean graphene has recently enabled the imaging of single molecules to about 8 Å resolution by low energy electron holography^33, 34^.

We present diffraction patterns from a limited number of aligned filaments, which exhibit well-resolved layer lines. In some cases, the diffraction patterns show asymmetric features that indicate the presence of a limited number of molecular rotations. Weak XFEL diffraction patterns can be oriented and merged in reciprocal space to further increase signal levels^31^. The high-resolution diffraction features in these merged patterns are better resolved than in conventional X-ray diffraction patterns. More generally, XFEL serial diffraction on graphene approaches the signal-to-noise levels needed to study single particles^35–37^, and thus shows promise as a practical method for the general study of amyloid fibrils and other weakly scattering particles of similar size.

## Results

### XFEL imaging of fibrils on free-standing graphene windows

**Fig. 1:**
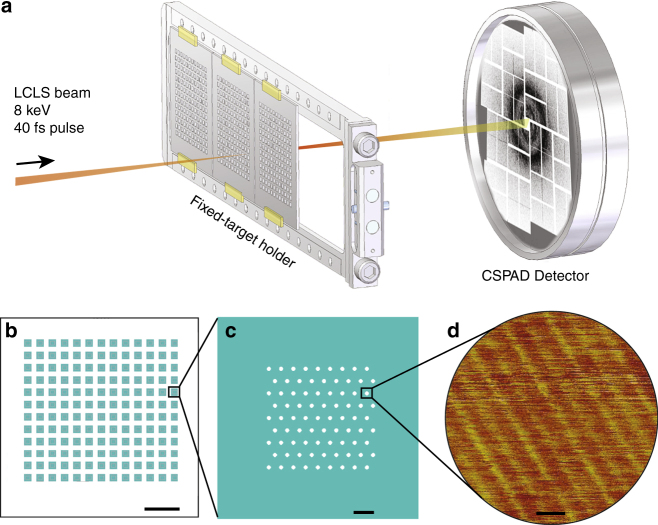
Experimental overview. **a** Silicon chips covered with a monolayer of graphene and a layer of fibrils were mounted in vacuum at the CXI beamline at LCLS and scanned through the XFEL focus. **b** Fixed-targets were made of a square silicon frame patterned with an array of 12 × 13 windows. Scalebar is 5 mm. **c** A single window contained 81 holes of either 20 or 30 μm in diameter arrayed on a hexagonal lattice, resulting in 12,636 holes over a 2.54 × 2.54 cm area. Scalebar is 100 μm. **d** An atomic force microscope image of a hole covered with graphene and fibrils is shown. Fibrils were imaged next to the hole on the silicon frame, as tapping without the support destroys the graphene layer. Scalebar is 20 nm

We used an ultraclean graphene layer placed on a holey silicon support frame to deliver non-crystalline filaments into the XFEL beam focus. Experiments were conducted in vacuum to minimize background scattering from air, and the X-ray beam was focused to a spot size of about 150 nm full-width at half maximum (FWHM) to maximize the flux incident on individual protofibrils. To further reduce other sources of background, low scattering silicon frames were engineered, as shown in Fig. 1a–c. The silicon frame was designed with robust, efficient and simple sample scanning in mind. The 20-µm diameter holes were an optimal balance between the visibility of holes in the on-axis microscope necessary to align the frame with the X-rays, reduction of the interaction between the wings of the focused beam with the chip, and preventing window membranes from breaking during fabrication. Holey frames were covered with a layer of ultraclean graphene. A fabrication process previously described for smaller free-standing graphene windows^33^ was modified as depicted in Supplementary Fig. 1 and detailed in Methods. Support frames were tested for graphene cleanliness, coverage, and stability of the graphene upon sample application using low energy electron and light microscopy (Supplementary Fig. 2). The cleanliness of the graphene windows was comparable to that described elsewhere^33, 38^.

Silicon frames with freshly prepared ultraclean graphene layers (with and without samples) were glued onto an aluminum frame prior to their introduction into the CXI^32, 39^ vacuum chamber. Data collection was performed using XFEL pulses of 40 fs duration at 8 keV photon energy and 1.5 mJ pulse energy at the beam focus, giving a calculated peak fluence of about 7 × 10^13^ photons/µm^2^. The experimental setup is depicted in Fig. 1a. The frames were scanned at 1.5 s^−1^ through the XFEL beam. This step scan was performed such that the XFEL pulse intersected every silicon hole, similar to previous fixed target approaches^40, 41^. Diffraction patterns were collected over two 24-hour shifts.

### Preparation of aligned filaments on graphene

We selected Tobacco Mosaic Virus (TMV) fibers as a reference sample, and functional hormone amyloid protofilaments prepared from bombesin and β-endorphin peptides^42^. TMV has a large asymmetric unit whose 3D structure has been determined to high-resolution by fiber diffraction and refined with cryo-EM^6, 43^. Soluble bombesin and β-endorphin act both as neurotransmitters in the central nervous system and control a wide spectrum of activities on the cell periphery, and bombesin has putative roles in cancer growth. Both hormones are arranged as amyloid fibrils inside secretory granules of cells^42^. In contrast to disease-related amyloid fibrils, hormone amyloid fibrils can disassemble into active peptides upon pH change, and they exhibit a very low degree of polymorphism, which is essential to this experiment^44^. Amyloid fibrils form and maintain their structure under extreme conditions including acidic environments and high temperatures^45^, and so are not expected to degrade in the vacuum in the XFEL chamber.

**Fig. 2:**
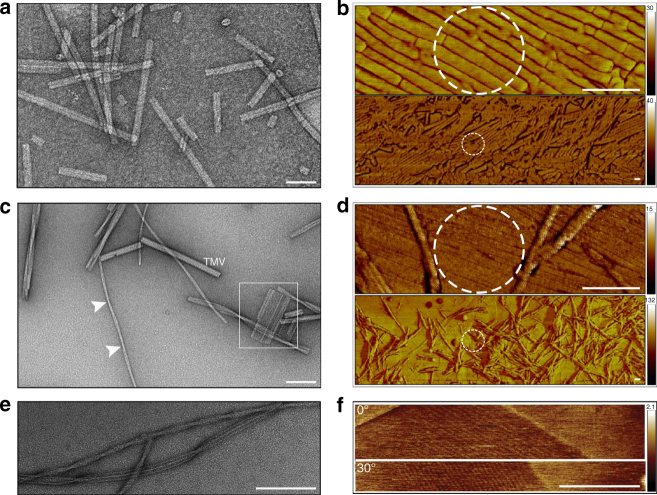
Preparation of TMV filaments and amyloid protofibrils on graphene. **a**, **b** Representative negative-stain TEM and AFM images of TMV, **c**–**d** bombesin filaments, and **e**, **f** β-endorphin filaments are shown. Negative-stain images (**a**,** c**,** e**) were acquired on fibrils placed on amorphous carbon films and AFM images (**b**,** d**,** f**) on graphene. Scalebars are 100 nm. **b** TMV fibrils align naturally on graphene over hundreds of nanometers. However, on the micrometer scale, aligned and randomly ordered fibrils are co-present. **c** Bombesin protofibrils associate laterally to form fibers, which randomly twist. A single preparation may consist of different polymorphs, e.g., twisted fibers and fibril rafts which are depicted here with arrows and squares, respectively. Bombesin fibers were mixed with TMV to compare their thickness. **d** The alignment of bombesin protofibrils on graphene is shown. Mature fibers are detected at larger magnifications. **e** β–endorphin protofibrils associate laterally to form twisted and striated fibers. **f** Aligned β–endorphin protofibrils were observed on graphene supports. To confirm that the features that are being imaged by the AFM are from the sample and not an artifact caused by the probe, the sample was rotated by 30**°** with respect to the scanning direction. Dashed circles represent the XFEL focus with FWHM = 150 nm

A key to obtaining useful diffraction signals from multiple filaments is their mutual alignment. Graphene provides a great benefit in this regard since it exhibits guiding forces to protein filaments, which tend to align them along their fibril axes^46, 47^. This effect can be clearly observed by comparing images of TMV (Fig. 2a, b), bombesin amyloid fibrils (Fig. 2c, d) and β-endorphin amyloid fibrils (Fig. 2e, f), when they are placed either on amorphous carbon films (Fig. 2a, c, e) or graphene (Fig. 2b, d, f). In contrast to TMV, amyloid fibers are visibly polymorphic and are composed of different numbers of protofibrils (Fig. 2c, e). We observed that individual protofibrils aligned with graphene (Fig. 2d, f), whereas the mature fibers did not.

Protofibrils were the targets of this experiment, and to initiate their formation we mixed purified peptide solutions of bombesin and β-endorphin with heparin at slightly acid pH values (pH 5.5) mimicking their native acidic environment in secretory granules^42^. Protofibril growth was imaged by negative-stain transmission electron microscopy (TEM) over four days (see Methods) and the existence of protofibrils was observed between 8–24 h after initiation of filamentation. Fibril suspensions were tested for alignment by depositing droplets on ultraclean graphene sheets dispersed on solid silicon. Atomic force microscopy (AFM) imaging showed that graphene appeared to stop assembly processes and maintain protofibril structures during the time of the AFM measurements (a few hours) (Fig. 2d, f). Protofibril dilution was calibrated to maximize the frequency of single layers.

β-endorphin protofibrils have an average diameter of 3 nm, which was identified from a one-dimensional intensity profile obtained from TEM images of straight fibers^44^. To estimate the diameter of bombesin protofibrils the signal-to-noise was increased by generating seven 2D class averages of fibers from the TEM micrographs (Supplementary Fig. 3). Pixel intensities in columns parallel to the fiber axis were summed to a one-dimensional profile, which revealed diameters of bombesin fibers ranging from 8.8 to 11.3 nm. Modulations in these intensity profiles (Supplementary Fig. 3b) suggest that bombesin fibers are composed of three to four protofibrils each with a width of 2–3 nm. Imaging TMV in the same micrograph shows that its diameter is about six times larger than that of the individual bombesin protofibrils (Fig. 2c, Supplementary Fig. 3).

### Scattering intensities of fibril and background components

**Fig. 3:**
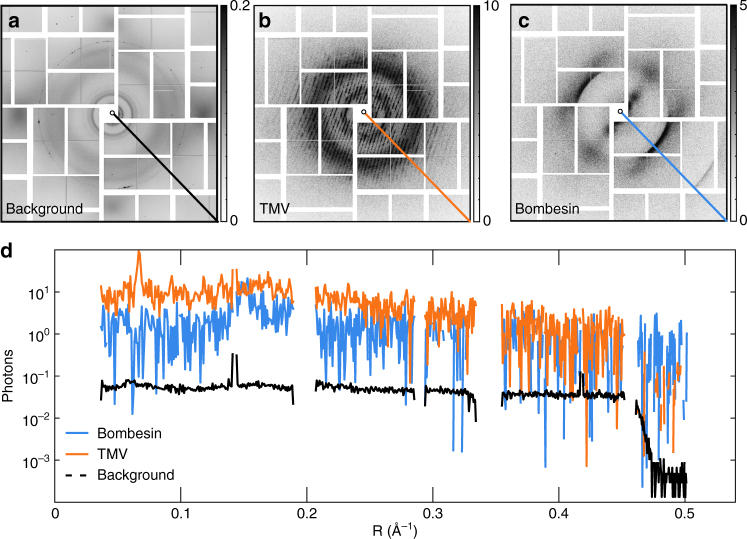
Diffraction images obtained at the LCLS in the CXI nanofocus chamber. **a** The average background from 1,607 selected frames with graphene but without sample. The diffuse scattering of the silicon and some contamination is visible. Single frames from **b** TMV and **c** the amyloid bombesin. **a**–**c** The grayscale shows photons per pixel. The average background contains 119,556 scattered photons, which is equivalent to about 0.050 photons/pixel (**a**). **d** Traces from the diagonal lines in (**a**–**c**) plotted as a function of reciprocal resolution R (Å^−1^). The average background of graphene-covered holes is two orders of magnitude lower than that due to the samples

A total of 126,768 diffraction frames were acquired from four dilutions of all three fiber types, on empty holes and holes covered with only graphene and no sample. To compare the scattering intensity from the fibril and graphene components and for calibrating background subtraction, we characterized the X-ray scattering from graphene-covered holes (Fig. 3) and sample-free, empty holes (Supplementary Fig. 4).

Frames showing diffraction from graphene-covered sample-free holes were selected from a series of 4352 detector frames with diluted TMV. The selection criteria are described in Supplementary Fig. 5 and Methods. The average background from sample-free holes contains about 119,556 scattered photons (Fig. 3a, Supplementary Fig. 4a). This is equivalent to about 0.05 photons/pixel. The scattering from empty holes was determined from a series of 1569 frames. Empty holes give rise to measurable diffuse scattering from the silicon chip (Supplementary Fig. 4b). The average total background per image from the series exposing only empty holes, excluding beam-off events, was about 101,345 scattered photons.

We find that scattering from empty holes is similar to that of graphene-covered sample-free holes, indicating that the main component of the average background (Fig. 3a, Supplementary Fig. 4a) is due to the empty holes alone. Additional background may contain contributions from misclassified very weak TMV hits (the fraction of patterns containing sample diffraction), as well as the graphene layer. Other sources of background seen in the difference (Supplementary Fig. 4c) between the average background (Fig. 3, Supplementary Fig. 4a) and the background from empty holes (Supplementary Fig. 4b) may be attributed to the parasitic scattering of the XFEL and iron fluorescence in the steel vacuum chamber^48^.

Hit fractions of 30–50% were achieved with samples that were diluted 20–250 times, starting with initial peptide concentrations of 1 mg/ml. Diffraction patterns from TMV exhibited layer lines in one or more orientations, indicating the presence of single or multiple layers of protofibrils in the nanofocus. An example diffraction frame from TMV with a single orientation on the graphene is shown in Fig. 3b. A pattern from bombesin is shown in Fig. 3c. Radial sections of these patterns are plotted in Fig. 3d. The signal from the amyloid and TMV are seen to extend to 2.4 Å and 2.7 Å, respectively. The background contribution from free-standing graphene is seen to be two orders of magnitude lower than the sample signal in Fig. 3d.

### Diffraction by TMV fibers

**Fig. 4:**
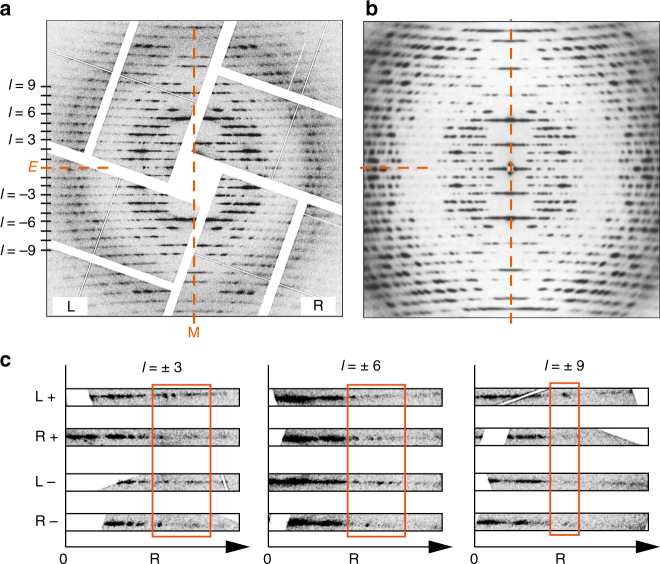
Comparison of XFEL and conventional X-ray fiber TMV diffraction patterns. **a** A single XFEL snapshot of TMV protofibrils on graphene is shown. The resolution at the center-edge is 3.86 Å. Left and Right layer lines, equatorial and meridional axes are labeled L, R, E, and M, respectively. **b** A classical X-ray fiber diffraction pattern from millions of mutually aligned TMV fibrils. Reprinted from publication^6^, Copyright (1989), with permission from Elsevier. **c** Magnifications of three symmetry related layer lines (*l* = ± 3, *l* = ± 6, *l* = ± 9) are shown as a function of resolution R. The left and right sides of the layer lines are indicated with L and R, and positive and negative layer lines with + and −, respectively. The left layer lines are flipped along the vertical axis to match the profile of the right layer lines

To demonstrate the structural integrity of the samples under our experimental conditions, we compare a single XFEL diffraction pattern from TMV exhibiting 24 layer lines (Fig. 4a) to a synchrotron diffraction pattern obtained from a specimen containing millions of TMV filaments aligned in well-oriented gels (Fig. 4b)^6^. The XFEL pattern resembles the azimuthally averaged synchrotron X-ray diffraction pattern. The qualitative agreement between the strong features suggests that the structure is not damaged in vacuum relative to the solvated form up to relatively high resolution. We selected 37 TMV XFEL frames with well-defined layer lines similar to Fig. 4a and calculated the period of the molecular structure along the *c* axis (fiber axis) from the layer line spacing. The average value is 68.8 Å, which agrees with the known value of 68.7 Å^43, 49^, and the values from individual patterns are equal to this value within the error bars (Supplementary Fig. 6). This suggests that the global structure of TMV is maintained during the experiment.

### Asymmetric features in single-shot XFEL diffraction patterns

A conventional fiber specimen contains many molecules with random axial rotations, and random directions either parallel or antiparallel to the fiber axis. A conventional fiber diffraction pattern is, therefore, cylindrically averaged, and so is symmetric about the equator (horizontal axis) and the meridian (vertical axis), as is evident in Fig. 4b. However, in some of the XFEL diffraction patterns, such as Fig. 4a, there is evidence that this symmetry is not present and there are observable differences along layer lines to the left and right of the meridian. Intensity profiles of the left and right halves of some of the layer lines are compared in Fig. 4c. The asymmetry indicates that the XFEL diffraction patterns from TMV are not cylindrically averaged, and that protofibrils with only one or a few axial rotations may be simultaneously exposed in the XFEL focus. Such patterns potentially contain more information than the cylindrically averaged patterns measured in conventional fiber diffraction experiments^50^.

Although the number of protofibrils within the focus is limited, their exact number is difficult to determine. The number of protofibrils was estimated from examination of tapping-mode AFM images of graphene-covered silicon next to the selected windows (similar to Fig. 2a–c) as the free-standing graphene is too fragile to withstand AFM analysis. Based on this analysis and the ~150 nm XFEL focal diameter, we estimate that fewer than about 50 amyloid protofibrils and about eight TMV fibers contributed to the single diffraction patterns shown in Fig. 3.

### Diffraction by amyloid protofibrils

**Fig. 5:**
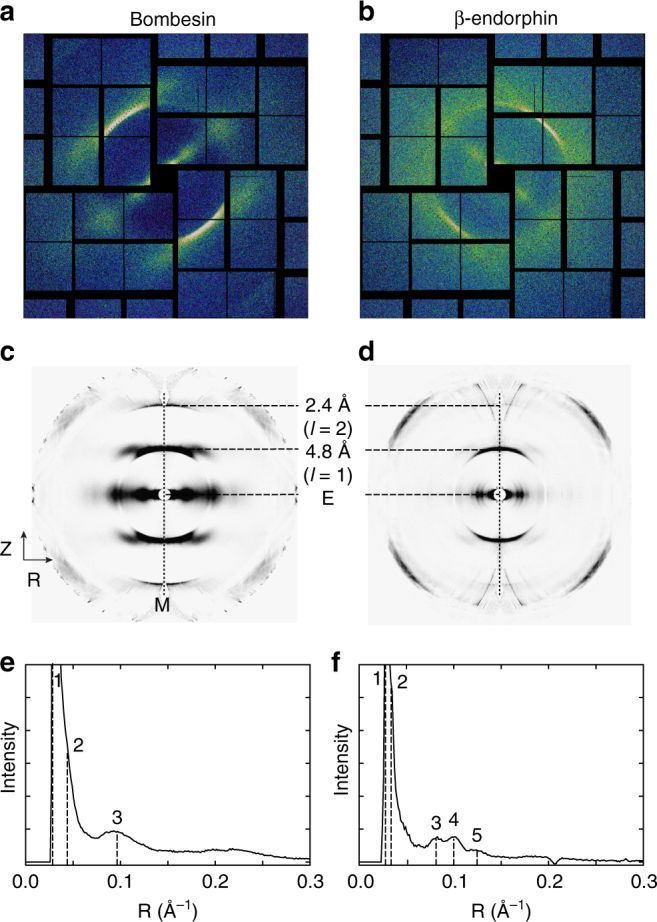
XFEL Diffraction patterns obtained from amyloid fibrils. Fibrils composed of bombesin and β-endorphin are shown on the left and right, respectively. **a**–**b** Single diffraction snapshots from aligned protofibrils, and background-subtracted merged patterns obtained from 40 diffraction snapshots each of **c** bombesin and **d** β-endorphin are shown. **e**,** f** Averaged intensity profiles as a function of reciprocal resolution over a band of width eight pixels ((**e**) bombesin) and 22 pixels ((**f**) β-endorphin) centered on the equator. Peaks in the equatorial profiles are marked. All peaks are summarized in Table 1

Amyloid protofibrils are about six times smaller in diameter than TMV particles (Fig. 2b and S3), and therefore exhibit broader diffraction features. Single diffraction snapshots from amyloid protofibrils of bombesin and β-endorphin are shown in Fig. 5a, b. These patterns exhibit strong intensity on the equator and a strong meridional layer line at about 4.8 Å due to the characteristic spacing of β-strands in β-sheets typical for amyloids^10^. This preserved c-repeat indicates that there are no global structural changes due to the experimental conditions. The second layer line at ~2.4 Å (4.8 Å / 2) on the meridian is also present in single snapshots.

Forty diffraction frames from each amyloid data set (bombesin and β-endorphin) with well-defined layer lines similar to Fig. 5a, b were selected manually, as existing hit-finding methods were found to be not suitable for detecting layer lines in the somewhat diffuse patterns of this kind. Patterns were aligned and registered in reciprocal space after their rotation angle around the beam axis (φ) and the deviation of the fiber axis from the normal to the beam axis (β) (Supplementary Fig. 7) were determined. The tilt angle between the fiber axis and the X-ray beam varied within a small range, independent of the substrate tilt due to buckling of graphene across the holes. The oriented frames were then mapped into reciprocal space (R, Z) for subsequent analysis, with coordinates normal (R), and parallel (Z), to the fiber axis^51^. The mapped patterns were merged, symmetrized and background-subtracted to give an averaged pattern in (R, Z) space with an improved signal-to-noise ratio (Fig. 5c, d). Averaged equatorial intensity profiles shown in Fig. 5e, f were used to determine positions of the equatorial maxima. For bombesin, three peaks including one pronounced equatorial peak at 10.6 Å are discernible (Figure 5e). β-endorphin fibrils show five peaks amongst which there are three pronounced maxima at 8.1 Å, 9.9 Å, and 12.3 Å labeled 3, 4, and 5, respectively (Fig. 5f). Both equatorial and meridional peaks are summarized in Table 1.

**Table 1:** Positions and qualitative intensities of diffraction maxima.

Peak	R, Å	1/R, Å^-1^	Intensity	Z, Å	1/Z, Å^-1^	Intensity
β-endorphin						
1	40.0	0.025	s	4.8	0.208	s
2	31.3	0.032	s	2.4	0.41	vw
3	12.3	0.081	s	—	—	—
4	9.9	0.101	s	—	—	—
5	8.1	0.123	s	—	—	—
bombesin						
1	34.5	0.029	s	4.8	0.208	s
2	23.8	0.042	s	2.4	0.41	w
3	10.6	0.094	s	—	—	—

R and Z positions were identified in averaged patterns of bombesin and β-endorphin (Fig. 5)

*s* strong, *w* weak, *vw* very weak

## Discussion

We have demonstrated a new approach to study non-crystalline amyloid fibrils combining femtosecond pulses from the LCLS XFEL and free-standing graphene windows. This approach presents two advantages: very low background scattering and mutual alignment of the particles in the beam focus. The average background scattering obtained of 0.1 photons/pixel approaches that obtained from aerosol injection methods for single particles using hard X-rays^48^ is significantly less than previously reported for other fixed target samples at LCLS^41, 52, 53^ and is dramatically lower than that obtained with a liquid jet injector^30^. By naturally aligning TMV filaments and protofibrils composed of bombesin and β-endorphin peptides, graphene fixes the alignment of the molecule during exposure. This new mounting scheme limits the number of filaments simultaneously exposed to the XFEL focus (about eight TMV filaments and less than 50 amyloid protofibrils) and with further development may allow data to be collected from single fibrils.

High-quality single-shot diffraction patterns were obtained from TMV. In some cases, asymmetry in the single-shot patterns indicates the presence of a limited number of axial rotations of the exposed particles. There are two implications of this observation. First, it suggests the possibility that the graphene may lock the TMV molecules into a specific rotation. Second, if the range of rotations present is small, then the data may represent a single, or a narrow, section through reciprocal space, rather than the cylindrical average. This is the case even if there is more than one molecule in the beam, as long as the molecules are mutually aligned. A full 3D data set could then be obtained from a range of fiber rotations in the x-ray beam. For molecules of high-order helix symmetry, the range of rotations required is small. For example, with TMV, a rotation range of 22 degrees would be sufficient. Such data could potentially be collected by tilting the support frame in the beam. With a full 3D data set from non-crystalline fibrils (i.e., 1D crystals), the information content is much higher than in a conventional cylindrically averaged fiber diffraction pattern, and direct, model-free phasing is feasible^54^.

**Fig. 6:**
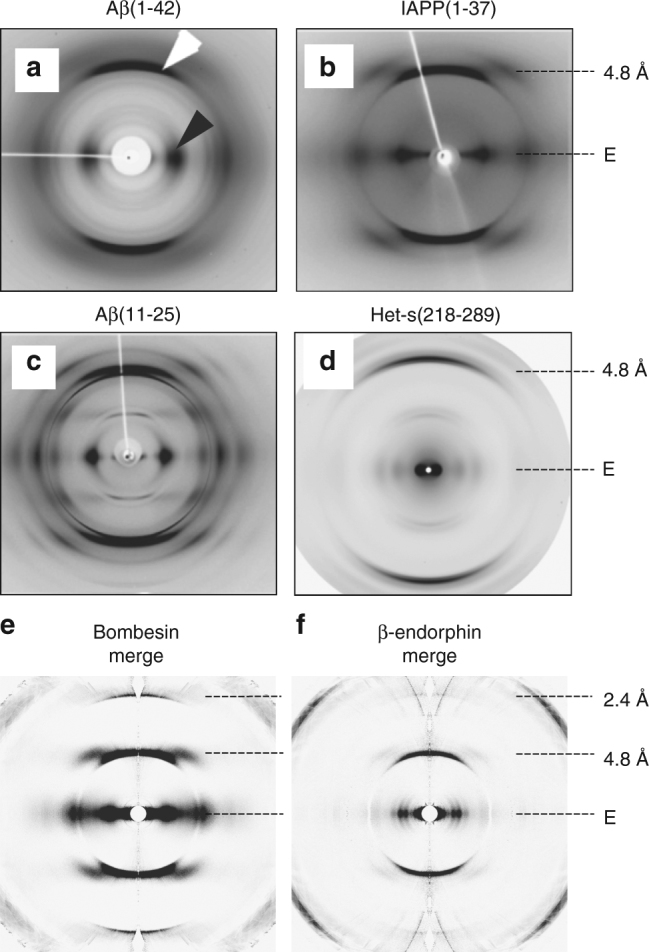
Comparison of conventional X-ray patterns to merged XFEL patterns. Diffraction patterns from amyloid fibers composed of **a** Aβ(1–42)^10^, **b** IAPP(1–37)^10^, **c** Aβ(11–25)^10^, and **d** Het-s(218-289)^58^ are shown. The equator and the most prominent layer lines are marked on the right side. The white and black arrow mark the meridional reflection at about 4.8 Å and the equatorial reflection at about ~10 Å characteristic for stacked β-sheets and present in (**a**–**d**), respectively. Note, that all amyloid fibrils are non-crystalline except for (**c**), which is crystalline. Merged XFEL diffraction patterns of bombesin (**e**) and β-endorphin fibrils (**f**) extending to 2.4 Å resolution are shown for qualitative comparison. **a**–**c** are reprinted from publication^10^, Copyright (2010), with permission from Elsevier. **d** is reprinted from publication^58^ (https://pubs.acs.org/doi/abs/10.1021%2Fbi5002807). Further permissions related to the material excerpted should be directed to the ACS

Snapshots from bombesin and β-endorphin protofibrils are of limited resolution, but they could be oriented and merged in reciprocal space to produce merged patterns with better signal-to-noise ratios and with an extended resolution. These merged patterns show reduced disorientation and background, and are of overall better clarity and higher resolution than those obtained from similar amyloids using synchrotron sources (Fig. 6).

The quality of the XFEL diffraction data obtained from the amyloid fibrils is limited due to the limited number of images and averaging of multiple protofibrils co-present in the focus. However, the strong equatorial peak at ~10 Å resolution in the XFEL diffraction pattern of bombesin (Fig. 5a, c, e) is consistent with amyloid models with two β-sheets laterally placed 10 Å apart^55^. The β-endorphin structure, which has three peaks at 8.1, 9.9, and 12.3 Å, is known from solid-state NMR data to be in a β-helix conformation^56, 57^. The distances of 8.1, 9.9, and 12.3 Å are in agreement with distances of opposing β-sheets in the fibril core. A similar equatorial intensity profiles was published for the β-solenoid structure of Het-s(218-289), with peaks about 17 and 11 (and 8 Å, not highlighted in the paper)^58^.

In the study reported here, a limited number of diffraction frames were collected as a result of the scanning system, which could only move the fixed target through the XFEL focus at 1.5 s^−1^. Newly available scanning hardware will be capable of keeping up with the 120 Hz repetition rate of the XFEL^59^. This will increase the data collection rate and with it the number of collected patterns and ultimately the quality of the merged data set.

The signal level obtained in single snapshots indicates that collection of data from single fibrils may be possible. In order to achieve this, biochemical methods must be developed to segregate single fibrils for exposure to the XFEL. For data collected from single fibrils, fixed rotations of the fibrils on the graphene is not necessary, as it is for the case of multiple fibrils in the beam described above. In fact, a variety of rotations will aid filling out 3D reciprocal space. A small range of fibril tilts (i.e., rotations about an axis normal to the fibril axis), which could be obtained by titling the support frame, would also be required to complete coverage of reciprocal space^31^. Such an approach will require development of computational techniques for auto-orientating the diffraction patterns (Supplementary Fig. 7). Data from single protofibrils should, therefore, allow reconstruction of the full 3D intensity distribution of a protofibril, similar to that recently demonstrated for crystalline fibrils^31^. Such 3D datasets potentially allow model-free structure determination as described above.

Our results indicate that serial fibril diffraction on graphene may become a practical method of for the study of very weakly scattering particles using XFEL diffraction. Protofibrils are not yet accessible in images from a cryogenic specimen, and high-resolution cryo-EM reconstructions from fibers, therefore, represent averages of multiple protofilament conformations^18, 19^. With this averaging both the high-resolution information of individual protofilaments and the conformational variability in flexible regions, which are likely to be of biological importance, are lost. In fact, there is a common consensus that it is not the amyloid fiber alone, but rather the protofilaments composing the fiber, and the process of fibril formation, that are toxic to the cell^22, 60^. XFEL-based experiments have the potential to overcome the challenges that come with a heterogenic specimen, such as a few nanometer thick amyloid fibrils. This represents a complementary tool to solid-state NMR and cryo-EM that has the potential to improve our understanding of individual protofilaments.

## Methods

### Design of silicon frames

Silicon frames with a size of 2.54 × 2.54 cm^2^ were commercially obtained from Norcada. Each frame contained 12,636 holes of either 20 or 30 μm in diameter arranged in an array of 12 × 13 square windows each 0.9 × 0.9 mm^2^, inside of which the silicon was thinned down to 2 μm. Individual windows contained 81 holes arrayed in a hexagonal layout with a pitch of 60 μm necessary for stability and for withstanding XFEL shock waves. The large hole sizes were necessary to reduce scattering by the edge of the hole from photons in the tails of the focused LCLS beam.

### Application of trivial transfer graphene on holey silicon

Graphene-PMMA in the form of trivial transfer graphene (TTG) was purchased from Advanced Chemicals Supplier (ACS). The graphene-PMMA was wet well with water before being cut with a scalpel to 2.54 × 2.54 cm^2^ and transferred to a deionized distilled water bath. The graphene-PMMA was transferred onto the silicon chip and allowed to dry on silicon at room temperature for 20–30 min. Polymethylmethacrylate (PMMA) was removed by immersing the silicon frame in an acetone bath. The silicon frame was placed on a hot plate at 300 °C for 2–4 h and covered with a silicon chip with a 50 nm thick metallic palladium layer, to catalyze the evaporation of residual PMMA^33^ (Supplementary Fig. 1).

### Peptide constructs

The amyloid samples presented in the manuscript were purchased from BACHEM. β-endorphin (human) trifluoroacetate salt with the sequence: YGGFMTSEKSQTPLVTLFKNAIIKNAYKKGE is available under the catalog number H-2700. Bombesin trifluoroacetate salt with the sequence pEQRLGNQWAVGHLM is available under the catalog number H-2155.

### Preparation of protofilaments

To initiate protofibril formation, peptide solutions of bombesin, and β-endorphin were mixed with heparin in water (pH 5.5) and allowed to assemble and grow under continuous stirring for four days. At different time points after initiation, between 5 min to 4 days, suspension droplets were deposited on carbon films and imaged by negative-stain transmission electron microscopy. Protofibrils were observed between 8–24 h after initiation of filamentation.

### Sample deposition on silicon frames

Immediately after Palladium-catalysis^33^, the fiber samples were applied on the frame. To ensure optimal coverage, single droplets of about 0.4 μl were applied to all windows. To increase the ratio of single layers of fibrils over many square micrometers, protofilaments were applied at five dilutions (1 × , 20 × , 50 × , 250 × and 1000 × ). After all drops were dried, the silicon frames covered with graphene and protofibrils were imaged by AFM or exposed to the XFEL beam.

### Atomic force microscopy imaging of fibrils

Images of aligned single fibril layers were acquired on graphene prepared on a silicon wafer, and on graphene placed on a holey silicon support. As free-standing graphene breaks upon contact with the cantilever and without a support underneath, images were acquired next to the graphene windows (on the silicon frame). AFM images were obtained with a Veeco XX in tapping mode. The cantilevers used (MPP-21120-10) were purchased from Bruker with a resonance frequency of 75 kHz and a spring constant of 3 nm^−^^1^.

### Mounting silicon chips for XFEL experiments

Silicon chips were mounted on aluminum frames supplied by LCLS using small slices of Kapton tape. All work was conducted in a clean-room environment and frames were prepared immediately before the beamtime to allow for the cleanest graphene surface achievable.

### X-ray data collection

Each window was shot once with a 40 fs XFEL pulse with a calculated focus of 150 nm at FWHM. The sample-detector distance was set to 85 mm, which gave a resolution of 1.6 Å in the outer corners of the detector with a photon energy of 8 keV. A total of 126,768 frames were recorded on the 2D Cornell-SLAC pixel array detector (CSPAD) in high gain mode^61^. Parasitic scattering was reduced using a post-sample tantalum aperture matching the silicon chip dimensions containing a 4.8 mm hole located immediately after the sample plane. The experiment was performed under proposal number LM27. The data is publicly available on the CXIDB^62 (ID 75)^ .

### Calculation of the average background

Frames containing diluted TMV sample were also collected and filtered for beam-off events (Supplementary Fig. 5). After events with no background (beam-off) were discarded, frames were classified in three groups based on the number of photons in the frame, the number of photons in a selected area of the frame expected to contain signal, and the number of photons in a selected area of the frame expected to have no signal. From this histogram, a total of 1607 frames were assigned to be sample-free and of signal level higher than that of empty holes.

### Transmission electron microscopy imaging of fibrils

Samples were adsorbed at 1×, 2×, or 5× dilutions as above for 40 s to thin carbon films that span a thick fenestrated 300 holes per copper grid. The grids were blotted, washed on two drops of deionized distilled water and negatively stained with 2% (w/v) uranyl formate (UF). The grids were imaged with a JEOL transmission electron microscope operating at 200 kV. Electron micrographs were recorded on a TVIPS TemCam F216 digital camera at a nominal ×50,000 magnification.

### Transmission electron microscopy and size determination

For TEM, stock solutions were diluted 2 × with D-PBS (Gibco Life Technologies). A volume of 4 μl of diluted samples were adsorbed for 60 s to glow-discharged parlodion carbon-coated copper grids. The grids were then blotted, washed on three drops of double-distilled water, incubated with 2 µl of Tobacco Mosaic Virus solution (TMV; kindly supplied by Ruben Diaz-Avalos, Janelia Research Campus, Ashburn, VA, USA), further washed with two drops of water and negatively stained with two drops of 2% uranyl acetate (pH 4.3) solution. Samples were imaged at a nominal magnification of ×52,000 using a Tecnai12 transmission electron microscope (FEI, Eindhoven, The Netherlands) operating at 120 kV. Electron micrographs were recorded on a 4000 × 4000 pixel charge-coupled device camera (F416, Tietz Video and Image Processing System, Gauting, Germany). Reference-free alignment was performed on manually selected fibril segments from recorded images using the EMAN2^63^ image processing package. A total of 488 segments of 128 × 128 pixels were extracted from the micrographs, aligned, and classified by multivariate statistical analysis yielding eight class averages: one of TMV and seven of the amyloid fibers. The TMV class average was aligned horizontally and the amyloid fiber class averages vertically by rotating the corresponding images. Density profiles were plotted using the Plot Profile tool from ImageJ^64^ and the apparent diameters of the fibrils were measured manually on the plots between the minima or deepest points. The estimated diameter of TMV was used to redetermine the recorded specimen area by each pixel (0.25 nm).

### Hit finding

Frames containing defined layer lines instead of arcs from the XFEL were manually selected using the Cheetah Software Suite^65^. Manual frame selection from these (comparatively) small datasets was used as existing hit-finding methods are not suitable for these kinds of patterns.

### Merging

Since single frames had a low degree of misorientation, the alignment and averaging was done manually. A custom graphical utility (Supplementary Fig. 7) was used in which the in-plane rotation angle φ and the out-of-plane tilt β are determined. The tilt was determined with the aid of horizontal and vertical guides, which were used to manually check that the layer lines are horizontal and that the non-equatorial peaks on opposite sides of the equator had the same radial coordinate. Four-quadrant averaging improved the signal-to-noise ratio and filled in the panel gaps in the detector. All selected frames were scaled and averaged. The background on the detector was assumed to be circularly symmetric except for the polarization effect. Since the signal was concentrated in layer lines, pixels from between the layer lines were used to calculate this symmetric background, which was then subtracted from the whole frame. The program used is available at https://github.com/kartikayyer/RZ-Gui.

### Code availability

Program is available at https://github.com/kartikayyer/RZ-Gui.

### Data availability

Other data are available from the corresponding author upon reasonable request.

## Electronic supplementary material

Supplementary Information

## References

[1] Stubbs G (1999). Developments in fiber diffraction. Curr. Opin. Struct. Biol..

[2] Millane RP (2010). X-ray fiber diffraction analysis. Int. Tables Crystallogr..

[3] Cochran W, Crick FHC, Vand V (1952). The structure of synthetic polypeptides .1. The transform of atoms on a helix. Acta Crystallogr.

[4] Watson JD, Crick FH (1953). Molecular structure of nucleic acids; a structure for deoxyribose nucleic acid. Nature.

[5] Marvin DA (1998). Filamentous phage structure, infection and assembly. Curr. Opin. Struct. Biol..

[6] Namba K, Pattanayek R, Stubbs G (1989). Visualization of protein-nucleic acid interactions in a virus. Refined structure of intact tobacco mosaic virus at 2.9 a resolution by x-ray fiber diffraction. J. Mol. Biol..

[7] Park HS, Arnott S, Chandrasekaran R, Millane RP, Campagnari F (1987). Structure of the alpha-form of poly[d(a)].Poly[d(t)] and related polynucleotide duplexes. J. Mol. Biol..

[8] Inouye H, Fraser PE, Kirschner DA (1993). Structure of beta-crystallite assemblies formed by alzheimer beta-amyloid protein analogues: Analysis by x-ray diffraction. Biophys. J..

[9] Sunde M (1997). Common core structure of amyloid fibrils by synchrotron x-ray diffraction. J. Mol. Biol..

[10] Jahn TR (2010). The common architecture of cross-beta amyloid. J. Mol. Biol..

[11] Wille H (2009). Natural and synthetic prion structure from x-ray fiber diffraction. Proc. Natl Acad. Sci. USA.

[12] Wan W (2015). Structural studies of truncated forms of the prion protein prp. Biophys. J..

[13] Tuttle MD (2016). Solid-state NMR structure of a pathogenic fibril of full-length human alpha-synuclein. Nat. Struct. Mol. Biol..

[14] Blake C, Serpell L (1996). Synchrotron x-ray studies suggest that the core of the transthyretin amyloid fibril is a continuous beta-sheet helix. Structure.

[15] Malinchik SB, Inouye H, Szumowski KE, Kirschner DA (1998). Structural analysis of alzheimer’s beta(1-40) amyloid: Protofilament assembly of tubular fibrils. Biophys. J..

[16] Walti MA (2016). Atomic-resolution structure of a disease-relevant abeta(1-42) amyloid fibril. Proc. Natl Acad. Sci. USA.

[17] Wasmer C (2008). Amyloid fibrils of the het-s(218-289) prion form a beta solenoid with a triangular hydrophobic core. Science.

[18] Fitzpatrick, A. W. P. et al. Cryo-em structures of tau filaments from alzheimer’s disease. *Nature ***547**, 185-190 (2017). 10.1038/nature23002 PMC5552202 28678775

[19] Gremer L (2017). Fibril structure of amyloid-beta(1-42) by cryo-electron microscopy. Science.

[20] Meier BH, Bockmann A (2015). The structure of fibrils from ‘misfolded’ proteins. Curr. Opin. Struct. Biol..

[21] Tycko R (2015). Amyloid polymorphism: Structural basis and neurobiological relevance. Neuron.

[22] Chiti F, Dobson CM (2006). Protein misfolding, functional amyloid, and human disease. Annu. Rev. Biochem.

[23] Henderson R (1995). The potential and limitations of neutrons, electrons and x-rays for atomic resolution microscopy of unstained biological molecules. Q. Rev. Biophys..

[24] Kendall A, Stubbs G (2006). Oriented sols for fiber diffraction from limited quantities or hazardous materials. J. Appl. Crystallogr.

[25] Cohen C, Harrison SC, Stephens RE (1971). X-ray diffraction from microtubules. J. Mol. Biol..

[26] Yamashita I, Suzuki H, Namba K (1998). Multiple-step method for making exceptionally well-oriented liquid-crystalline sols of macromolecular assemblies. J. Mol. Biol..

[27] Chapman HN (2006). Femtosecond diffractive imaging with a soft-x-ray free-electron laser. Nat. Phys..

[28] Neutze R, Wouts R, van der Spoel D, Weckert E, Hajdu J (2000). Potential for biomolecular imaging with femtosecond x-ray pulses. Nature.

[29] Chapman HN (2011). Femtosecond x-ray protein nanocrystallography. Nature.

[30] Popp, D. et al. Flow-aligned, single-shot fiber diffraction using a femtosecond x-ray free-electron laser. *Cytoskeleton* *(Hoboken)* **74**, 472-481 (2017). 10.1002/cm.21378 28574190

[31] Wojtas, D. H. et al. Analysis of xfel serial diffraction data from individual crystalline fibrils. *IUCrJ* **4**, 795-811 (2017). 10.1107/S2052252517014324 PMC5668865 29123682

[32] Liang MN (2015). The coherent x-ray imaging instrument at the linac coherent light source. J. Synchrotron Radiat..

[33] Longchamp, J. N. Ultraclean freestanding graphene by platinum-metal catalysis. *J. Vac. Sci. Technol. B* **31**, 020605-1-020605-3 (2013).

[34] Longchamp JN (2017). Imaging proteins at the single-molecule level. Proc. Natl Acad. Sci. USA.

[35] Hantke MF (2016). A data set from flash x-ray imaging of carboxysomes. Sci. Data.

[36] Ekeberg T (2016). Single-shot diffraction data from the mimivirus particle using an x-ray free-electron laser. Sci. Data.

[37] Bogan MJ (2008). Single particle x-ray diffractive imaging. Nano. Lett..

[38] Longchamp JN, Escher C, Latychevskaia T, Fink HW (2014). Low-energy electron holographic imaging of gold nanorods supported by ultraclean graphene. Ultramicroscopy.

[39] Boutet, S. & Williams, G. J. The coherent x-ray imaging (cxi) instrument at the linac coherent light source (lcls). *New. J. Phys*. **12**, 035024 (2010).

[40] Frank M (2014). Femtosecond x-ray diffraction from two-dimensional protein crystals. IUCrJ.

[41] Hunter MS (2014). Fixed-target protein serial microcrystallography with an x-ray free electron laser. Sci. Rep..

[42] Maji SK (2009). Functional amyloids as natural storage of peptide hormones in pituitary secretory granules. Science.

[43] Sachse C (2007). High-resolution electron microscopy of helical specimens: a fresh look at tobacco mosaic virus. J. Mol. Biol..

[44] Seuring, C. et al. Amyloid fibril polymorphism: almost identical on the atomic level, mesoscopically very different. *J. Phys. Chem. B*. **121**, 1783-1792 (2017). 10.1021/acs.jpcb.6b10624 28075583

[45] Wang L, Schubert D, Sawaya MR, Eisenberg D, Riek R (2010). Multidimensional structure-activity relationship of a protein in its aggregated states. Angew. Chem. Int. Ed. Engl..

[46] Lo VC, Pham QRCLL, Morris VK, Kwan AH, Sunde M (2014). Fungal hydrophobin proteins produce self-assembling protein films with diverse structure and chemical stability. Nanomaterials.

[47] Svaldo-Lanero T (2012). Aligning amyloid-like fibrils on nanopatterned graphite. BioNanoScience.

[48] Munke, A. et al. Coherent diffraction of single rice dwarf virus particles using hard x-rays at the linac coherent light source. *Sci. Data* **3**, 160064 (2016). 10.1038/sdata.2016.64 PMC4968191 27478984

[49] Kendall A, McDonald M, Stubbs G (2007). Precise determination of the helical repeat of tobacco mosaic virus. Virology.

[50] Namba K, Stubbs G (1987). Isomorphous replacement in fiber diffraction using limited numbers of heavy-atom derivatives. Acta Crystallogr. Sect. A.

[51] Stribeck N, Nochel U (2009). Direct mapping of fiber diffraction patterns into reciprocal space. J. Appl. Crystallogr.

[52] Feld GK (2015). Low-z polymer sample supports for fixed-target serial femtosecond x-ray crystallography. J. Appl. Crystallogr.

[53] Pedrini B (2014). 7 Å resolution in protein two-dimensional-crystal x-ray diffraction at linac coherent light source. Philos. Trans. R. Soc. Lond. B. Biol. Sci..

[54] Millane RP (2017). The phase problem for one-dimensional crystals. Acta Crystallogr. Sect. A Found. Adv..

[55] Perutz MF, Finch JT, Berriman J, Lesk A (2002). Amyloid fibers are water-filled nanotubes. Proc. Natl Acad. Sci. USA.

[56] Gath, J. *Amyloid fibrils seen by solid-state nmr: Structure, dynamics, interactions*, ETH Zurich, (2013).

[57] Verasdonck, J. *Solid-state nmr Observations Of Amyloid Fibrils: A Journey From Spin Space To Real Space* (ETH Zurich, Zurich, 2017).

[58] Wan W, Stubbs G (2014). Fiber diffraction of the prion-forming domain het-s(218-289) shows dehydration-induced deformation of a complex amyloid structure. Biochemistry.

[59] Roedig, P. et al. A micro-patterned silicon chip as sample holder for macromolecular crystallography experiments with minimal background scattering. *Sci. Rep*. **5**, 10451 (2015). 10.1038/srep10451 PMC4448500 26022615

[60] Haass C, Selkoe DJ (2007). Soluble protein oligomers in neurodegeneration: Lessons from the alzheimer’s amyloid beta-peptide. Nat. Rev. Mol. Cell. Biol..

[61] Herrmann S (2013). Cspad-140k: A versatile detector for lcls experiments. Nucl. Instrum. Methods Phys. Res. A.

[62] Maia FRNC (2012). The coherent x-ray imaging data bank. Nat. Methods.

[63] Ludtke SJ, Baldwin PR, Chiu W (1999). Eman: semiautomated software for high-resolution single-particle reconstructions. J. Struct. Biol..

[64] Schneider CA, Rasband WS, Eliceiri KW (2012). Nih image to imagej: 25 years of image analysis. Nat. Methods.

[65] Barty A (2014). Cheetah: Software for high-throughput reduction and analysis of serial femtosecond x-ray diffraction data. J. Appl. Crystallogr.

