# Supplementary material for: Femtosecond X-ray coherent diffraction of aligned amyloid fibrils on low background graphene
Source: Nat Commun. 2018 May 9;9:1836. doi: 10.1038/s41467-018-04116-9 (PMC5943278; doi:10.1038/s41467-018-04116-9)
Supplement: Supplementary file 1 — Supplementary Information [file 41467_2018_4116_MOESM1_ESM.pdf]

Supplementary Information

Femtosecond X-ray coherent diffraction of aligned amyloid fibrils on low background graphene

Seuring et. al

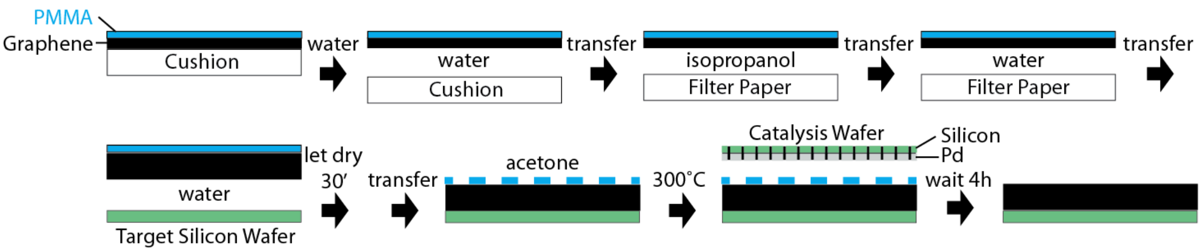

Supplementary Figure 1. A flowchart describing the preparation of a holey silicon wafer with free-standing graphene windows (Figure 1) starting with trivial transfer graphene on a cushion. In comparison to a previous method[1], ultraclean graphene was prepared after an initial acetone wash with a second holey silicon wafer covered with a layer of palladium. By sandwiching PMMA between the target silicon chip and this catalysis wafer, the deposition of a strong scatterer (Palladium) on the sample wafer was avoided.

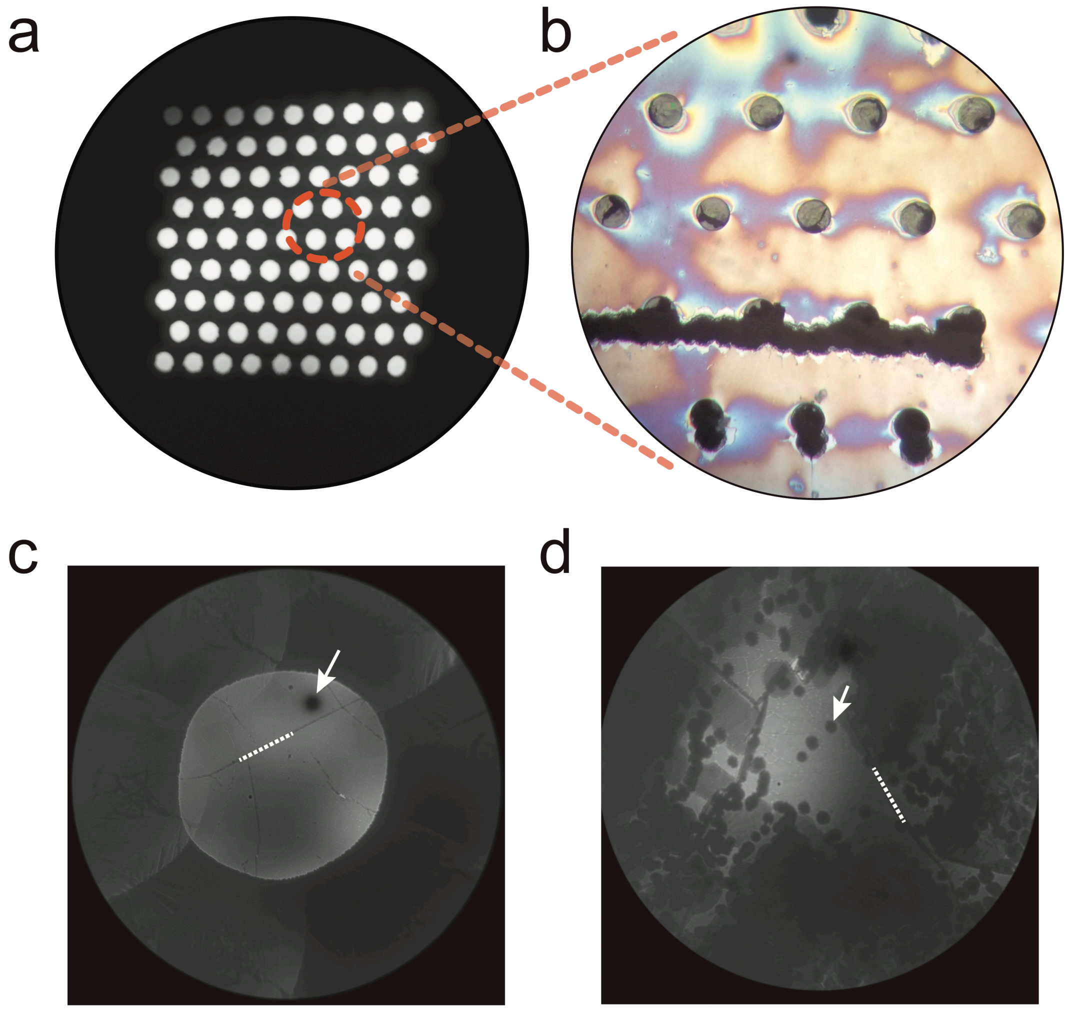

Supplementary Figure 2. Quality check of free-standing graphene windows. (a) A photograph of one window of a silicon chip with 81 empty holes is depicted (Figure 1). Holes are 20 µm in diameter. (b) A light microscopy image of magnified holes covered with graphene and a layer of fibrils after exposure to the XFEL. The damage by the XFEL pulse has a size similar to the size of the hole. The XFEL pulses hit between the holes when misaligned, but the damage did not propagate. Low-energy microscopy images of graphene free-standing over (c) 5 µm and (d) 20 µm holes after Palladium-catalysis. Gray areas of a few µm^2 are ultraclean. From these images, we estimate that about 30-50% of the free-standing area is ultraclean. Dotted lines and arrows show graphene grain-boundaries and aborted carbon grains, respectively.

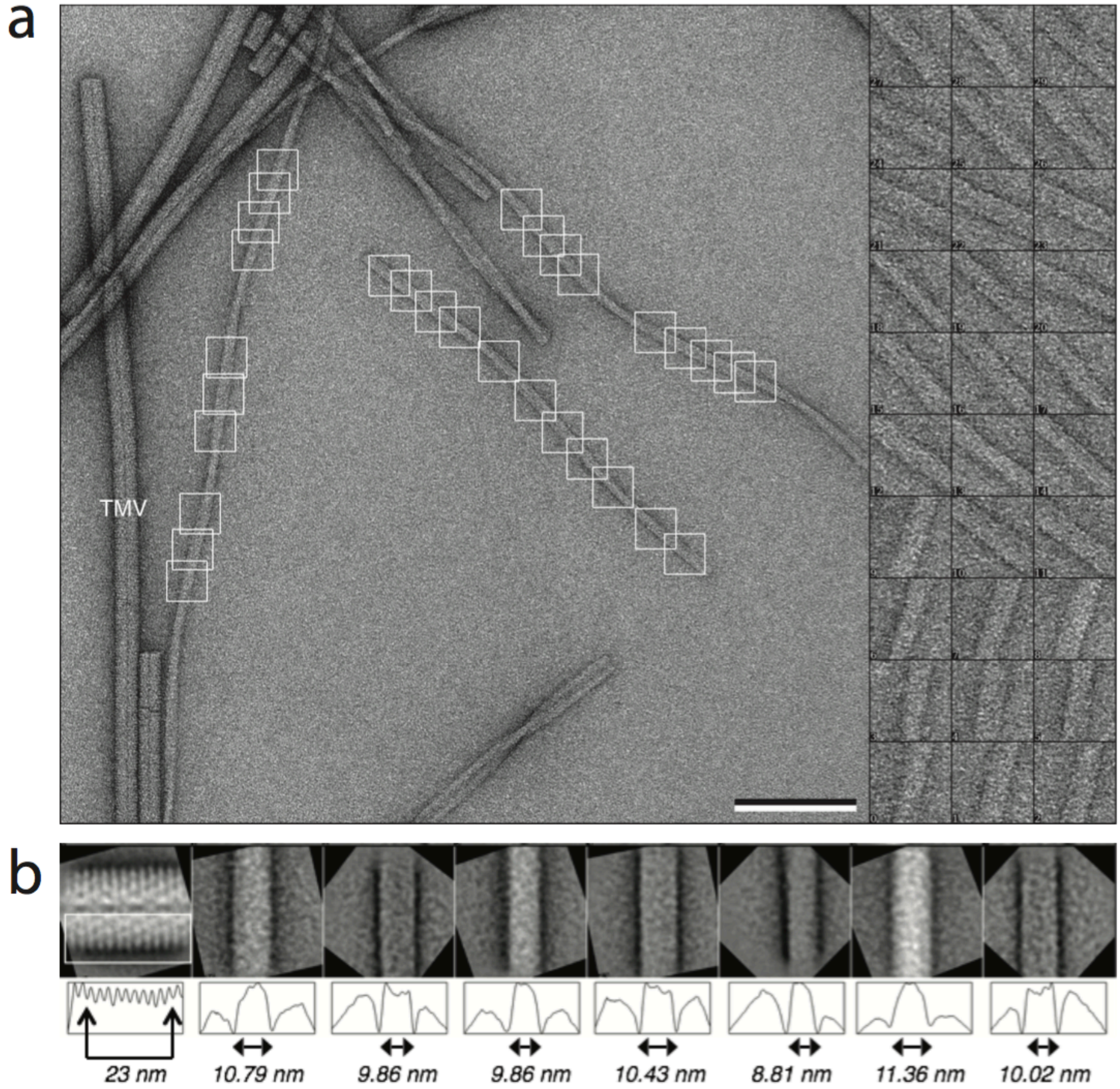

Supplementary Figure 3. Analysis of the width of bombesin fibers. (a) Negative-stain TEM images of mature bombesin fibers composed of protofibrils. TMV was included as a reference. Bombesin fibers vary in diameter and are often twisted. Fibril segments were analysed using sub-images of 32 x 32 nm^2. The scalebar is 100 nm. (b) A total of 355 sub-images of fiber segments were averaged in seven classes to measure seven fiber intensity profiles. The 23 nm distance corresponds to 10 turns of the TMV helix.

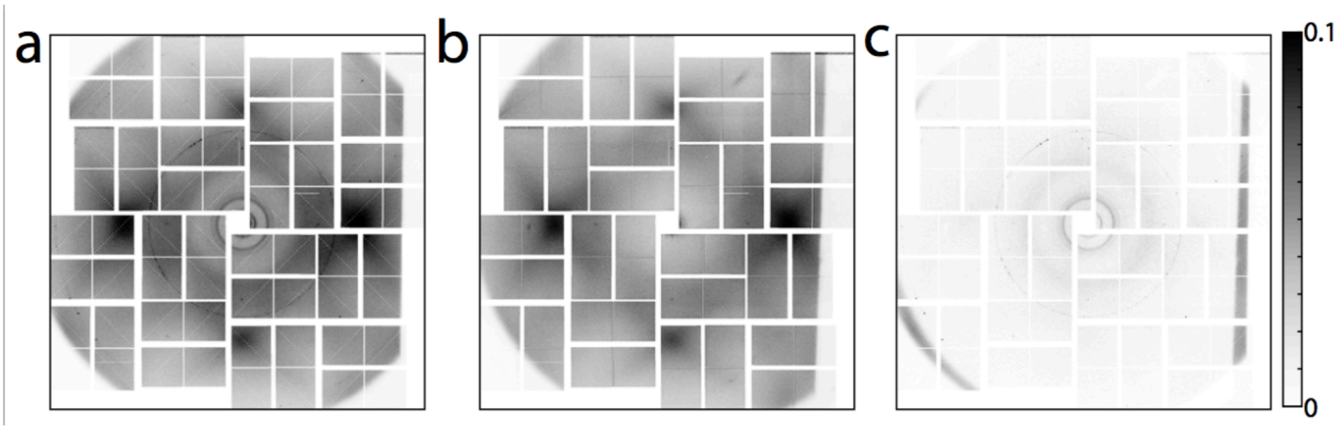

Supplementary Figure 4. Holes in silicon give rise to diffuse scattering. The average background diffraction from (a) 1,607 frames of holes covered with graphene but no fibers, and (b) from 1,569 frames of naked holes are shown. (c) The difference between (a) and (b) suggests that the main source of the background diffraction is interference from the wings of the XFEL pulse with the silicon holes and possible slight misalignment of the XFEL beam with the holes.

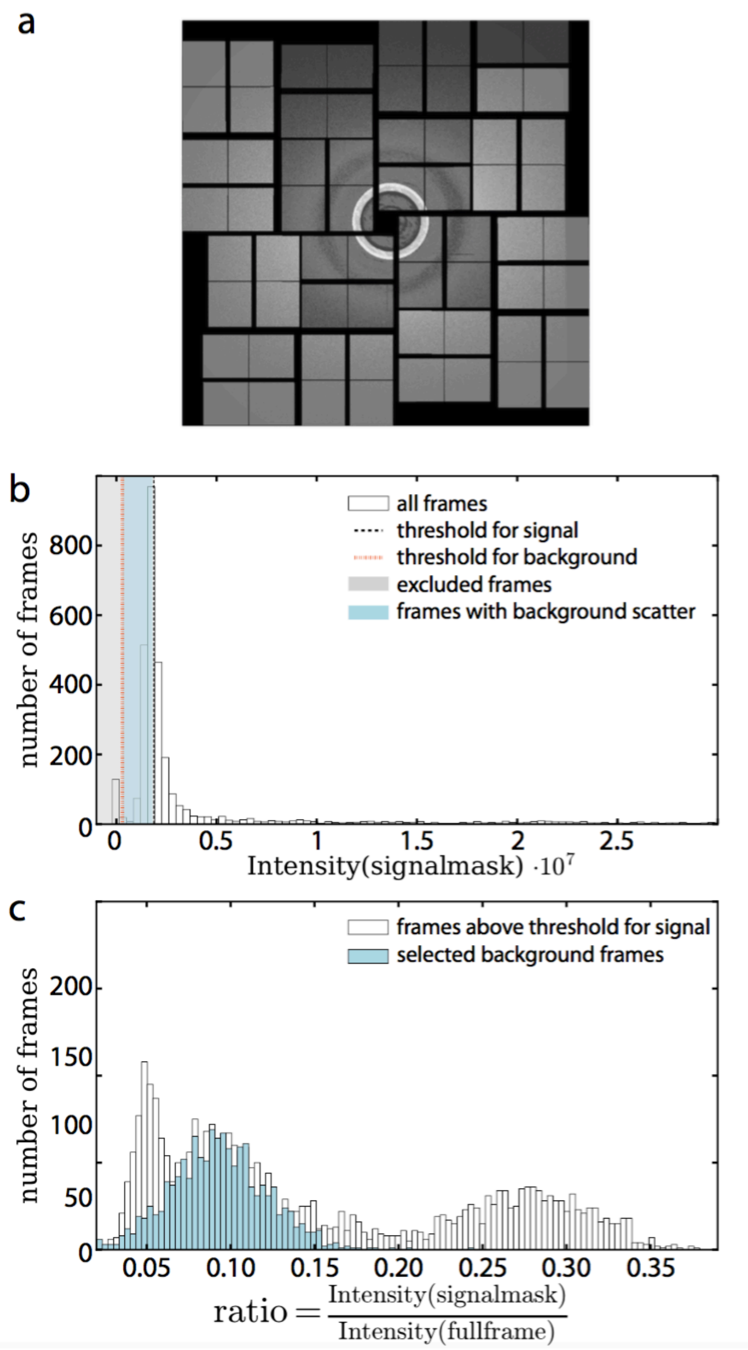

Supplementary Figure 5. The process of selecting frames without signal from a run with sample is depicted. (a) Frames containing sample scatter from TMV exhibit signal within the white circle. Intensity values per frame within the circular mask were used to generate a histogram (b). A threshold was applied to reject beam-off events. (c) Selected frames with intensities above the threshold were then grouped by the ratio of the intensity within the mask to the intensity of the whole frame. Three peaks represent groups of empty holes, background events and events with sample. Background events were averaged to determine the mean background to subtract from each image.

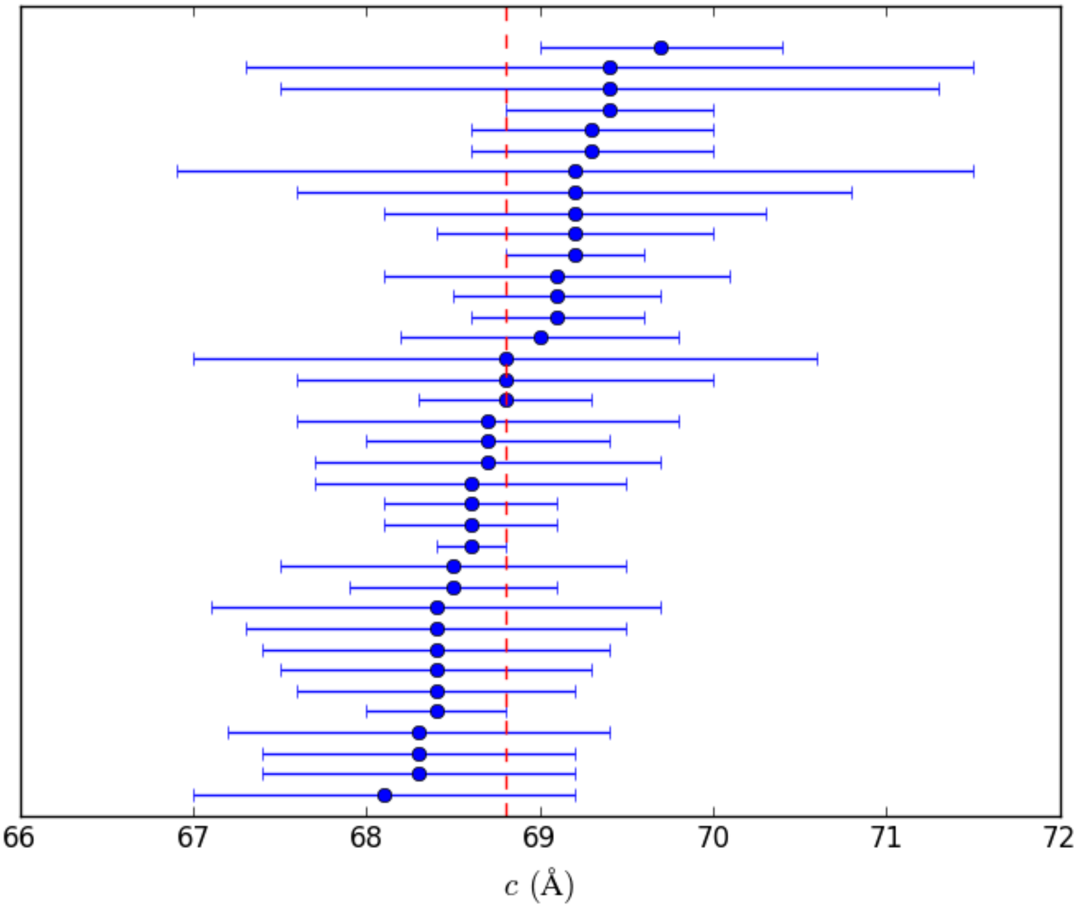

Supplementary Figure 6. C-repeats measured from 37 TMV diffraction patterns. Each bar shows the mean (filled circle) +/- 1 standard deviation. The average value of 68.7 Å is shown by the red dashed line[2,3]. Frames are ordered by the mean-value (red line).

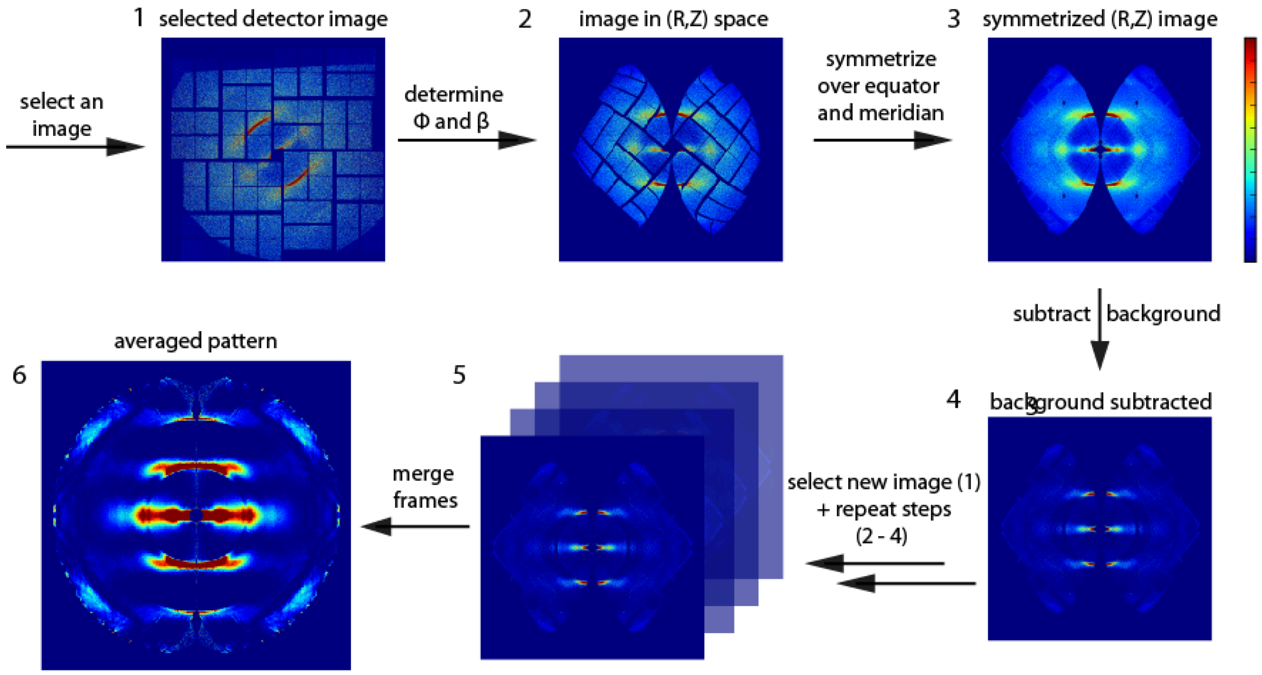

Supplementary Figure 7. Flow-chart depicting the assembly of multiple frames to a merged diffraction pattern. The colorbar shows increasing intensity, blue to red. (1) A single snapshot. (2) The four-fold symmetry of the layer lines is used to assign the rotation angle around the beam axis (ϕ) and the deviation of the fiber axis from the normal to the beam axis (β). These values are used to map the pattern into reciprocal space (R,Z) with coordinates normal (*R*), and parallel (*Z*) to the fiber axis[4]. (3) The pattern is symmetrized about the equator and the meridian, and an angularly symmetric background is subtracted (4) (see Methods). (5) This procedure is repeated with multiple oriented diffraction images, which are then merged to a single frame with a higher resolution signal (6).

Supplementary References

1 Longchamp, J. N. Ultraclean freestanding graphene by platinum-metal catalysis. (2013).

2 Kendall, A., McDonald, M. & Stubbs, G. Precise determination of the helical repeat of tobacco mosaic virus. *Virology* **369**, 226-227, (2007).

3 Sachse, C. *et al.* High-resolution electron microscopy of helical specimens: A fresh look at tobacco mosaic virus. *J. Mol. Biol.* **371**, 812-835, (2007).

4 Stribeck, N. & Nochel, U. Direct mapping of fiber diffraction patterns into reciprocal space. *J. Appl. Crystallogr.* **42**, 295-301, (2009).
